# Evaluating Imaging Biomarkers of Acquired Resistance to Targeted EGFR Therapy in Xenograft Models of Human Head and Neck Squamous Cell Carcinoma

**DOI:** 10.3389/fonc.2018.00271

**Published:** 2018-07-23

**Authors:** Lauren C. J. Baker, Arti Sikka, Jonathan M. Price, Jessica K. R. Boult, Elise Y. Lepicard, Gary Box, Yann Jamin, Terry J. Spinks, Gabriela Kramer-Marek, Martin O. Leach, Suzanne A. Eccles, Carol Box, Simon P. Robinson

**Affiliations:** ^1^Division of Radiotherapy & Imaging, The Institute of Cancer Research, London, United Kingdom; ^2^Division of Cancer Therapeutics, The Institute of Cancer Research, London, United Kingdom

**Keywords:** HNSCC, EGFR, resistance, imaging, MRI, PET

## Abstract

**Background:** Overexpression of EGFR is a negative prognostic factor in head and neck squamous cell carcinoma (HNSCC). Patients with HNSCC who respond to EGFR-targeted tyrosine kinase inhibitors (TKIs) eventually develop acquired resistance. Strategies to identify HNSCC patients likely to benefit from EGFR-targeted therapies, together with biomarkers of treatment response, would have clinical value.

**Methods:** Functional MRI and ^18^F-FDG PET were used to visualize and quantify imaging biomarkers associated with drug response within size-matched EGFR TKI-resistant CAL 27 (CAL^R^) and sensitive (CAL^S^) HNSCC xenografts *in vivo*, and pathological correlates sought.

**Results:** Intrinsic susceptibility, oxygen-enhanced and dynamic contrast-enhanced MRI revealed significantly slower baseline ${\text{R}}_{2}{}^{*}$, lower hyperoxia-induced $\Delta {\text{R}}_{2}{}^{*}$ and volume transfer constant K^trans^ in the CAL^R^ tumors which were associated with significantly lower Hoechst 33342 uptake and greater pimonidazole-adduct formation. There was no difference in oxygen-induced ΔR_1_ or water diffusivity between the CAL^R^ and CAL^S^ xenografts. PET revealed significantly higher relative uptake of ^18^F-FDG in the CAL^R^ cohort, which was associated with significantly greater Glut-1 expression.

**Conclusions:** CAL^R^ xenografts established from HNSCC cells resistant to EGFR TKIs are more hypoxic, poorly perfused and glycolytic than sensitive CAL^S^ tumors. MRI combined with PET can be used to non-invasively assess HNSCC response/resistance to EGFR inhibition.

## Introduction

Head and neck squamous cell carcinoma (HNSCC) is a significant cause of morbidity and mortality, with almost 700,000 new cases estimated annually, worldwide (1). For locally advanced head and neck cancer (LAHNC), the standard of care is chemoradiotherapy (with/without initial surgery) (2). Even with aggressive multimodality management, the majority of patients with LAHNC develop incurable locoregional or systemic relapse (3). For patients with recurrent and/or metastatic (R/M) disease, prognosis is poor and survival rates are dismal, highlighting a requirement for novel therapeutic options together with clinically useful predictive biomarkers (4).

The epidermal growth factor receptor (EGFR) belongs to the ErbB/HER family of transmembrane receptor tyrosine kinases and has a key role in HNSCC (5). Ligand binding to EGFR activates many downstream signaling pathways, promoting cell proliferation, survival, DNA repair, migration, invasion and angiogenesis (6). Elevated expression of EGFR occurs in >80% HNSCC, and is associated with poor prognosis and treatment resistance (7, 8). EGFR is thus a compelling therapeutic target, and a number of EGFR family antagonists have been developed (5, 6). The monoclonal antibody cetuximab has an established role in combination with radiotherapy in newly-diagnosed LAHNC (9), and with cisplatin/5-FU in R/M disease (10). Clinical responses to first generation EGFR tyrosine kinase inhibitors (TKIs), such as gefitinib or erlotinib, in HNSCC were disappointing (11). However afatinib, an irreversible HER family inhibitor, recently demonstrated improved progression-free survival in patients with R/M HNSCC (12). Nevertheless, the overwhelming majority of patients who initially respond to targeted therapies only achieve stable disease of short duration and acquired resistance usually manifests within 6–12 months (13–15).

A clinical definition of acquired resistance to EGFR TKIs has been proposed for patients with non-small cell lung cancer (16). These guidelines are based on detecting known drug-sensitizing *EGFR* mutations, combined with anatomical imaging, to detect disease progression whilst on therapy. However, in HNSCC, this approach is not applicable, since *EGFR* mutations are rare (17). Furthermore, levels of EGFR protein do not correlate with clinical response to EGFR TKIs in HNSCC (13, 18). Alternative strategies to identify HNSCC patients who are likely to benefit from EGFR-targeted therapies, together with biomarkers of response for monitoring therapy, are urgently required (19, 20).

An additional challenge in the treatment of the often bulky HNSCC tumors is hypoxia. The low levels of oxygen that result from an anarchic tumor vasculature and uncontrolled cell division are a well-established cause of treatment resistance and adversely affect HNSCC prognosis (21, 22). Under hypoxic conditions, altered gene transcription and adaptive signaling networks enable cancer cells to resist apoptosis and continue to proliferate in adversity, leading to the development of an increasingly aggressive tumor (23, 24). Expression and activation of EGFR are moderated by hypoxia although the mechanisms involved are yet to be fully elucidated (25). Co-localization of hypoxic regions and EGFR expression has been demonstrated in HNSCC biopsies and was associated with poor outcome (26). Conversely, hypoxic areas of HNSCC tumors were found to have reduced EGFR expression (27), possibly due to degradation of EGFR by the hypoxia-induced protein PHD-3 (28), and this was proposed as a putative mechanism of resistance to anti-EGFR therapies.

For the assessment of molecularly targeted agents such as TKIs, the standard response evaluation criteria in solid tumors (RECIST) may be suboptimal (29). This is particularly pertinent in the context of drug resistance, where genomic and morphological transformations typically precede changes in tumor volume. Advances in imaging technologies provide a means of defining non-invasive quantitative biomarkers to inform on biologically relevant structure-function relationships in tumors, enabling their accurate detection, an understanding of their behavior, and informing on response to targeted and, by extension, conventional treatments, at relatively early time points (30). Predictive imaging biomarkers of sensitivity and resistance to targeted anti-cancer treatments would thus provide opportunities to deliver personalized and more effective therapy regimes (31, 32).

To investigate EGFR TKI resistance in HNSCC, we have generated a CAL 27 cell line, CAL^R^, that is resistant to multiple EGFR TKIs (gefitinib, erlotinib, lapatinib and afatinib) (33). An isogenic control cell line, CAL^S^, retained sensitivity to EGFR TKIs *in vitro* and *in vivo* (33). The aim of this study was to exploit CAL^R/S^ xenografts to identify clinically translatable functional imaging biomarkers that (i) correctly report on the pathology and processes relevant to acquired resistance to EGFR inhibition in HNSCC *in vivo* and (ii) may have value in assessing response to targeted EGFR inhibition and, potentially, conventional radiotherapy/chemotherapy. To this end, a range of advanced magnetic resonance imaging (MRI) and positron emission tomography (PET) techniques, and the quantitative imaging biomarkers they afford, were evaluated and pathologically qualified to assess tumor hypoxia, angiogenesis and metabolism *in vivo*.

## Materials and methods

### Cell culture and tumor propagation

EGFR TKI-resistant CAL^R^ and -sensitive CAL^S^ HNSCC cells were cultured in Dulbecco's modified Eagle's medium supplemented with 10% fetal calf serum (Invitrogen, Paisley, UK) and maintained at 37°C in a humidified incubator with an atmosphere of 95% air, 5% CO_2_ (33). Cells were routinely screened for mycoplasma using PCR and had STR profiles (obtained using a GenePrint® 10 kit, Promega, Southampton, UK and a 3730xl DNA analyser, Applied Biosystems, Warrington, UK) identical to parental CAL 27 cells. STR profiles were obtained contemporaneous to the *in vivo* experiments (October 2013) and re-checked after completion (August 2015).

All animal experiments were performed in accordance with the local ethical review panel, the UK Home Office Animals (Scientific Procedures) Act 1986, the United Kingdom National Cancer Research Institute guidelines for the welfare of animals in cancer research (34), and the ARRIVE (Animal Research: Reporting of *In Vivo* Experiments) guidelines (35). Female NCr-*Foxn1*^*nu*^ mice (7–8 weeks old, Charles River) were injected with either 5 × 10^5^ CAL^R^ or 5 × 10^6^ CAL^S^ cells subcutaneously in the right flank. Ten-fold fewer CAL^R^ cells were used to compensate for the more rapid tumor growth rate of the resulting xenografts *in vivo* (33). Animals were housed in specific pathogen-free rooms in autoclaved, aseptic microisolator cages with a maximum of four animals per cage. Food and water were provided *ad libitum*. The mice were routinely monitored for the appearance of palpable tumors and imaged at a tumor diameter of ~0.8 to 1 cm.

### MRI data acquisition

MRI was performed on a 7 T horizontal bore microimaging system (Bruker, Ettlingen, Germany) using a 3 cm birdcage volume coil. Anaesthesia was induced with an intraperitoneal injection of fentanyl citrate (0.315 mg ml^−1^) plus fluanisone [10 mg ml^−1^ (Hypnorm; Janssen Pharmaceutical Ltd., High Wycombe, UK)], midazolam (5 mg ml^−1^ [Hypnovel; Roche, Welwyn Garden City, UK)], and sterile water (1:1:2). A lateral tail vein was cannulated with a 27G butterfly catheter (Hospira, Royal Leamington Spa, UK) for remote administration of contrast media. Mice were positioned in the coil on a custom-built platform to isolate the tumor, and with a nosepiece for gas delivery, and their core temperature maintained at 37°C with warm air blown through the magnet bore.

Multiple contiguous 1 mm thick axial T_2_-weighted images were acquired for localization and subsequent quantitation of the tumor volume. Magnetic field homogeneity was optimized by shimming over the entire tumor volume using an automated shimming routine (FASTmap).

For intrinsic susceptibility and oxygen-enhanced MRI, multiple gradient recalled echo (MGRE) images [repetition time (TR) = 200 ms, 8 echo times (TE) ranging from 6 to 28 ms, 4 ms echo spacing, 8 averages, acquisition time (AQ) 2 min 30 s], and inversion recovery (IR) TrueFISP images (TE = 1.2 ms, TR = 2.4 ms, 25 inversion times spaced 155 ms apart, initial inversion time of 109 ms, total scan TR = 10 s, α = 60°, 8 averages, AQ of 8 min) were acquired from three contiguous 1 mm slices across the center of the tumor over a 3 × 3 cm field of view (FOV) and 128 × 128 matrix whilst the host breathed air, to enable quantitation of the transverse relaxation rate ${\text{R}}_{2}{}^{*}$ (s^−1^), and the longitudinal relaxation rate R_1_ (s^−1^), respectively. The gas supply was then switched to 100% oxygen administered at 1 l min^−1^ and, following a 5 min transition time, identical MGRE and IR-TrueFISP image sets were acquired (36).

Dynamic contrast enhanced (DCE-) MRI data were acquired from a single 1 mm thick, central axial slice across the tumor using an inversion recovery (IR) true-FISP sequence with one baseline scan (3 × 3 cm field of view, 128x96 matrix, T_I_ = 25–1451 ms, 50 inversion times, T_R_ = 2.4 ms, T_E_ = 1.2 ms, 8 averages), and 60 dynamic scans (T_I_ = 109–924 ms, 8 inversion times, T_R_ = 2.4 ms, T_E_ = 1.2 ms, scan T_R_ = 10 s, 1 average, 60° flip angle, temporal resolution = 20 s) acquired for 3 min before and 17 min after an i.v. injection of 0.1 mmol kg^−1^ of a clinically approved, low molecular weight contrast agent gadolinium-DTPA (Gd-DTPA, Magnevist, Schering, Berlin, Germany) (37). A single slice acquisition was used for the IR-true-FISP sequence in order to minimize the temporal resolution of the dynamic acquisition and thereby optimize the accuracy of pharmacokinetic parameter estimates (38). The imaging slice used herein was taken through the largest axial extent of the tumor and assumed to be representative of the tumor as a whole.

Diffusion weighted (DW) images were acquired from the same three 1 mm thick axial slices as for intrinsic susceptibility and oxygen-enhanced MRI using an echo-planar imaging (EPI) sequence (3 × 3 cm FOV, 128 × 128 matrix, T_R_ = 3,000 ms, 5 b values ranging from 40 to 700 s^−1^mm^2^, 4 averages).

### MRI data analysis

Tumor volumes were determined using segmentation from regions of interest (ROIs) drawn on T_2_-weighted images for each tumor-containing slice. Functional MRI data were fitted on a pixel-by-pixel basis using in-house software (Imageview, developed in IDL, ITT Visual Information Systems, Boulder, CO, USA). MGRE, IR-TrueFISP and DW images were fitted using a Bayesian maximum *a posteriori* approach, allowing estimates of the oxygen-induced change in ${\text{R}}_{2}{}^{*}(\Delta {\text{R}}_{2}{}^{*})$ ($\Delta {\text{R}}_{2}{}^{*}$) and R_1_ (ΔR_1_), and the median ADC to be calculated, respectively (39, 40). For the IR-TrueFISP data, voxels with calculated T_1_ values < 200 or >3,000 ms were excluded from the analysis. For DCE-MRI analysis, IR true-FISP data were fitted using a similar approach, utilizing the dual-relaxation sensitivity (T_1_ and T_2_) of the pulse sequence and incorporating the Tofts and Kermode pharmacokinetic model, providing estimates of K^trans^ (37). In addition, model-free analysis was used to derive the initial area under the gadolinium uptake curve (IAUGC_60_, mM Gd min^−1^) from 0 to 60 s after injection of Gd-DTPA, and the ratio of enhancing to total tumor volume (enhancing fraction; EF, %).

### ^18^F-fluorodeoxyglucose (FDG) PET

Following overnight fasting, mice bearing CAL^R^ or CAL^S^ tumors were continuously warmed using a heating pad prior to and during the tracer uptake period. Both fasting and warming reduce normal/brown fat tissue radiotracer uptake, thereby enhancing tumor contrast (41). Mice were intravenously administered with 6.2–6.5 MBq of ^18^F-FDG (Alliance Medical Radiopharmacy Ltd, Sutton, UK) 30 min after initiation of warming, and PET-CT imaged 1 h later under isoflurane anesthesia using a trimodal PET/SPECT/CT scanner (Carestream Health, Rochester, NY, USA) for a duration of 600 s. A calibration phantom containing ~0.5–1.5 MBq ^18^F-FDG was positioned in the middle of the FOV and imaged simultaneously to ensure accurate quantification of tumor tracer uptake. The total activity in the FOV was < 10 MBq, where acceptable linearity of the PET scanner has previously been established (42). Each PET scan was followed by a CT examination (45 kVp, 0.4 mA, 250 projections) for attenuation correction and anatomical localisation.

PET images were reconstructed with a maximum likelihood expectation maximization algorithm using 12 iterations and a voxel size of 0.5 mm^3^. Scatter, randoms and decay correction were applied within the reconstruction with attenuation correction derived from the CT data. The CT data was reconstructed with filtered back projection. The PET/CT images were co-registered and analyzed using PMOD (version 3.501, PMOD Technologies Ltd, Zurich, Switzerland). Whole tumor ROIs were delineated using an isocontour method based on 50% of the maximum tumor uptake value, and the uptake of ^18^F-FDG quantified by calculation of the mean percentage injected dose per gram (%ID/g) using PMOD, corrected using the calibration phantom data.

### Histopathological assessment of tumor perfusion, hypoxia, glucose transporter-1, and cellular density

Mice bearing CAL^R^ or CAL^S^ tumors were administered the hypoxia marker pimonidazole (60 mg kg^−1^ i.p., Hypoxyprobe Inc., Burlington, VT, USA) and the perfusion marker Hoechst 33342 (15 mg kg^−1^ i.v., Sigma-Aldrich, Poole, UK), 45 min and 1 min, respectively, prior to necropsy. Tumors were rapidly excised and bisected in the imaging plane at the position of the central MRI slice, with half the tumor snap-frozen over liquid nitrogen and the other half formalin-fixed and paraffin-embedded (FFPE).

Fluorescence signals from reduced pimonidazole adducts bound with mouse monoclonal FITC-conjugated antibodies (Hypoxyprobe Inc.) and from Hoechst 33342 were detected and quantified in whole tumor frozen sections (10 μm) using a motorized scanning stage (Prior Scientific Instruments, Cambridge, UK) attached to a BX51 microscope (Olympus Optical, London, UK), driven by image analysis software (CellP, Soft Imaging System, Münster, Germany), as previously described (37). Immunohistochemical (IHC) detection of glucose transporter 1 (Glut-1) expression was performed on FFPE sections (5 μm), as previously described (43). In addition to a composite whole tumor image, high magnification (x100) images were acquired from ten randomly selected fields for each tumor, from which the percentage of Glut-1 staining was determined using color deconvolution in ImageJ (NIH, Bethesda, MD, USA). Cellular density was assessed on haematoxylin and eosin (H&E) stained FFPE sections by counting the number of nuclei in four square ROIs from a total area of 0.01 mm^2^ per field (x200 magnification, six fields assessed per section) (44).

### Statistical analysis

Statistical analysis of the MRI, PET and histopathological data was performed with GraphPad Prism ver 6.07 (GraphPad Software, La Jolla, CA, USA). Cohort sizes used for each experiment are shown in each figure (N.B. each animal did not necessarily undergo all the functional MRI scans). Results are presented in the form of mean ± 1 s.e.m. Following application of a Shapiro-Wilk normality test to confirm the Gaussian distribution of the data, significance testing employed either unpaired Student's two-tailed *t*-test assuming equal variance or the non-parametric Mann-Whitney test as appropriate, with a 5% level of significance.

## Results

HNSCC xenografts derived from EGFR TKI-resistant CAL^R^ cells grew more rapidly than CAL^S^ tumors, as previously reported (33). There was however no significant difference in the mean tumor volumes (as determined by T_2_-weighted MRI) of the CAL^R^ (533 ± 45 mm^3^) and CAL^S^ (506 ± 79 mm^3^) cohorts at the time of imaging.

**Figure 1:**
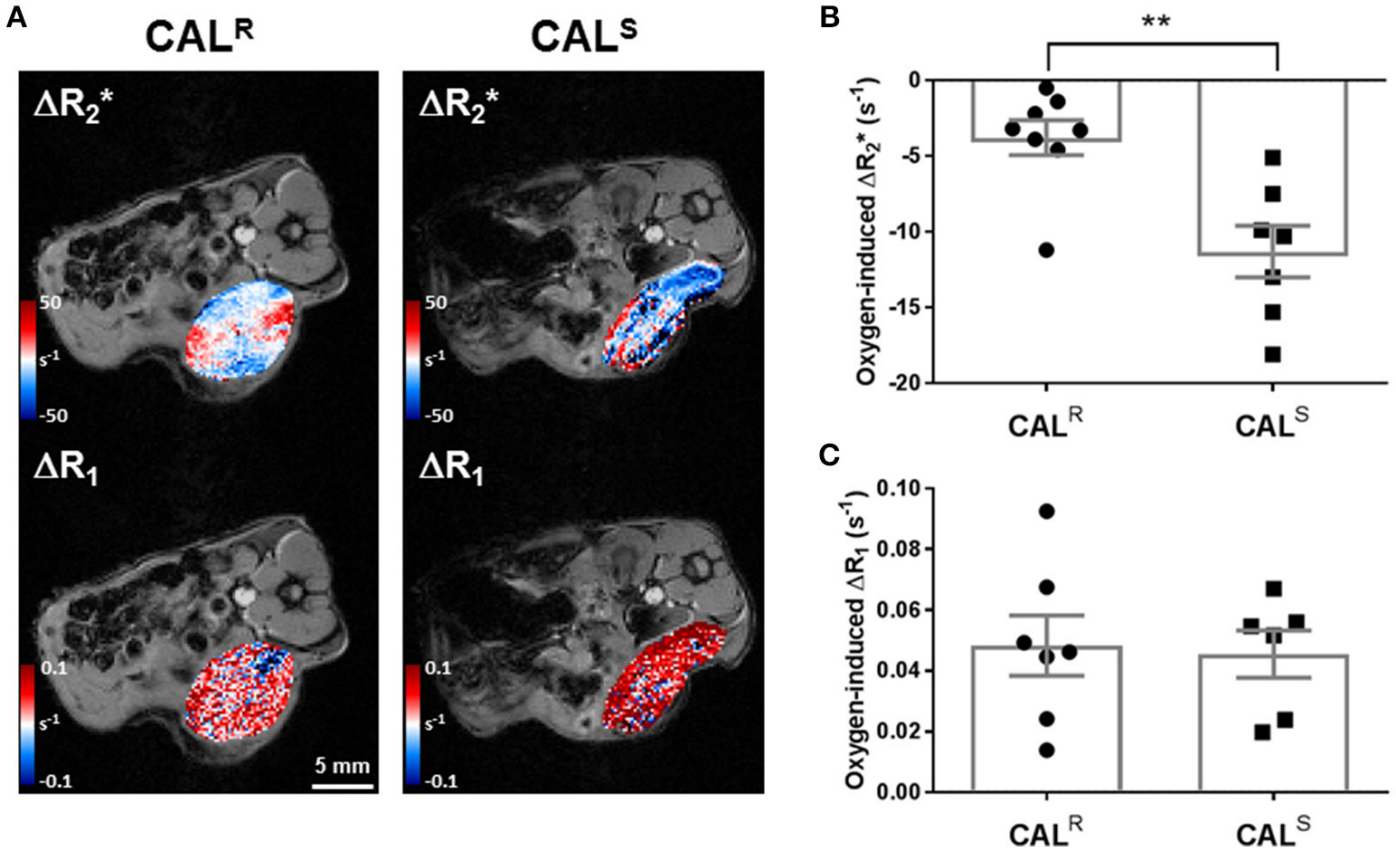
**(A)** Oxygen-induced ΔR_2_* and ΔR_1_ (both s^−1^) parametric maps acquired from a CAL^R^ and a CAL^S^ HNSCC xenograft *in vivo*. **(B,C)** Summary of the quantitative oxygen-enhanced MRI data, showing a significantly greater hyperoxia-induced ΔR_2_* in the CAL^S^ relative to the CAL^R^ cohort. Data are the individual oxygen-induced ΔR_2_* and ΔR_1_ measurements for each tumor, and the cohort mean ± 1 s.e.m., **p < 0.01, Student's *t*-test.

A hyperoxia-induced reduction in tumor ${\text{R}}_{2}{}^{*}$, resulting from the flushing out of paramagnetic deoxyhaemoglobin with diamagnetic oxyhaemoglobin, and altered tumor R_1_ arising from more dissolved paramagnetic oxygen in blood, are being actively investigated for the provision of imaging biomarkers of hypoxia (36, 45). Parametric maps showing the spatial distribution of oxygen-induced $\Delta {\text{R}}_{2}{}^{*}$ and ΔR_1_ in a CAL^R^ and a CAL^S^ tumor are shown in Figure 1A. Visually, hyperoxia predominantly induced a heterogeneous reduction in ${\text{R}}_{2}{}^{*}$ in both cohorts, which was greater in the CAL^S^ tumors. Overall, a small, heterogeneous oxygen-induced increase in R_1_ was the predominant response in both tumor types. No spatial relationship between hyperoxia-induced $\Delta {\text{R}}_{2}{}^{*}$ and ΔR_1_ was apparent in either tumor type. Quantitatively, mean baseline ${\text{R}}_{2}{}^{*}$ was significantly (*p* < 0.01) slower in the CAL^R^ (58 ± 2 s^−1^) than the CAL^S^ (70 ± 2 s^−1^) tumors. Oxygen-inhalation resulted in an overall reduction in mean ${\text{R}}_{2}{}^{*}$ in both CAL^R^ (−4 ± 1 s^−1^) and CAL^S^ (−11 ± 2 s^−1^) tumors, the response of the CAL^S^ tumors being significantly (*p* < 0.01) greater than that of the CAL^R^ xenografts (Figure 1B). There was no significant difference in mean baseline R_1_ (0.49 ± 0.003 and 0.49 ± 0.007 s^−1^) and mean hyperoxia-induced ΔR_1_ (0.05 ± 0.01 and 0.05 ± 0.01 s^−1^) between the CAL^R^ and CAL^S^ tumors, respectively (Figure 1C).

**Figure 2:**
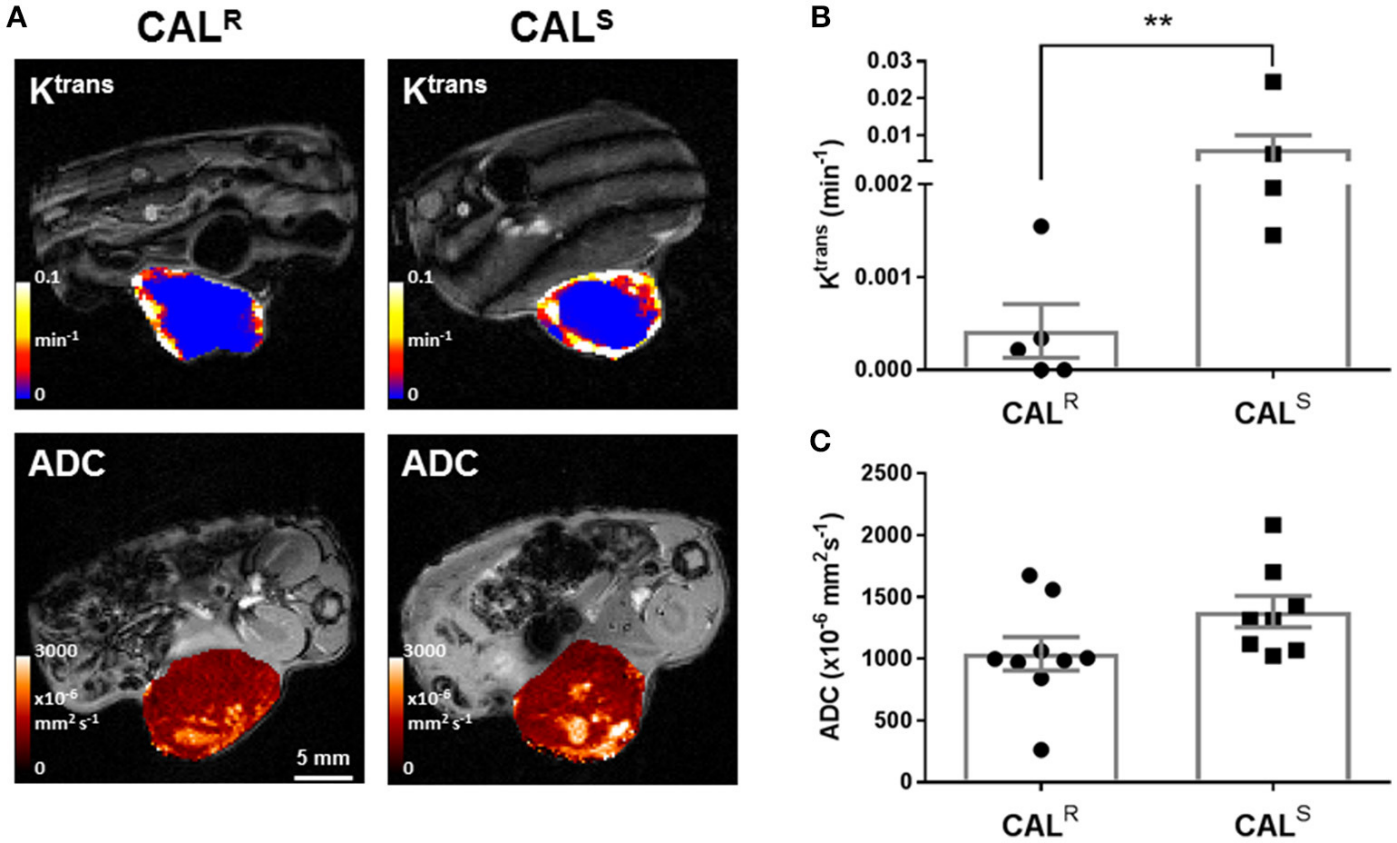
**(A)** Parametric maps of the transfer constant K^trans^ (min^−1^) and the apparent diffusion coefficient, ADC (x10^−6^ mm^2^s^−1^), acquired from different CAL^R^ and CAL^S^ HNSCC xenografts *in vivo*. **(B,C)** Summary of the quantitative DCE- and DWI MRI data, showing a significantly lower K^trans^ in the CAL^R^ relative to the CAL^S^ cohort. Data are the individual K^trans^ and ADC measurements for each tumor, and the cohort mean ± 1 s.e.m., **p < 0.01, Mann-Whitney test.

DCE-MRI measures contrast agent extravasation from the blood plasma compartment to the extravascular extracellular compartment, i.e., vascular leakage, typically expressed by the volume transfer constant K^trans^ (min^−1^). Increasing contrast agent concentration in the extracellular leakage space is related to both tumor perfusion and permeability (46). DCE-MRI revealed that Gd-DTPA delivery was restricted to the periphery of both CAL^R^ and CAL^S^ xenografts (Figure 2A). Pharmacokinetic modeling of the DCE-MRI data revealed that K^trans^ was significantly (*p* < 0.01) lower in the CAL^R^ tumors (Figure 2B). Model free analysis showed similarly significant lower IAUGC_60_ (0.0006 ± 0.0004 vs. 0.010 ± 0.005 mM Gd min^−1^, *p* < 0.01) and EF (51 ± 6 vs. 82 ± 4%, *p* < 0.01) in the CAL^R^ xenografts.

DW-MRI exploits the random diffusion of water molecules to measure differences in tissue cellularity, quantified through the measurement of the apparent diffusion coefficient (ADC, x10^6^ mm^2^s^−1^) (47). Parametric ADC maps from both CAL^R^ and CAL^S^ tumors were generally homogeneous, but with discrete regions of markedly elevated water diffusion (Figure 2A). There was no significant difference in mean ADC between the two tumor types (Figure 2C).

**Figure 3:**
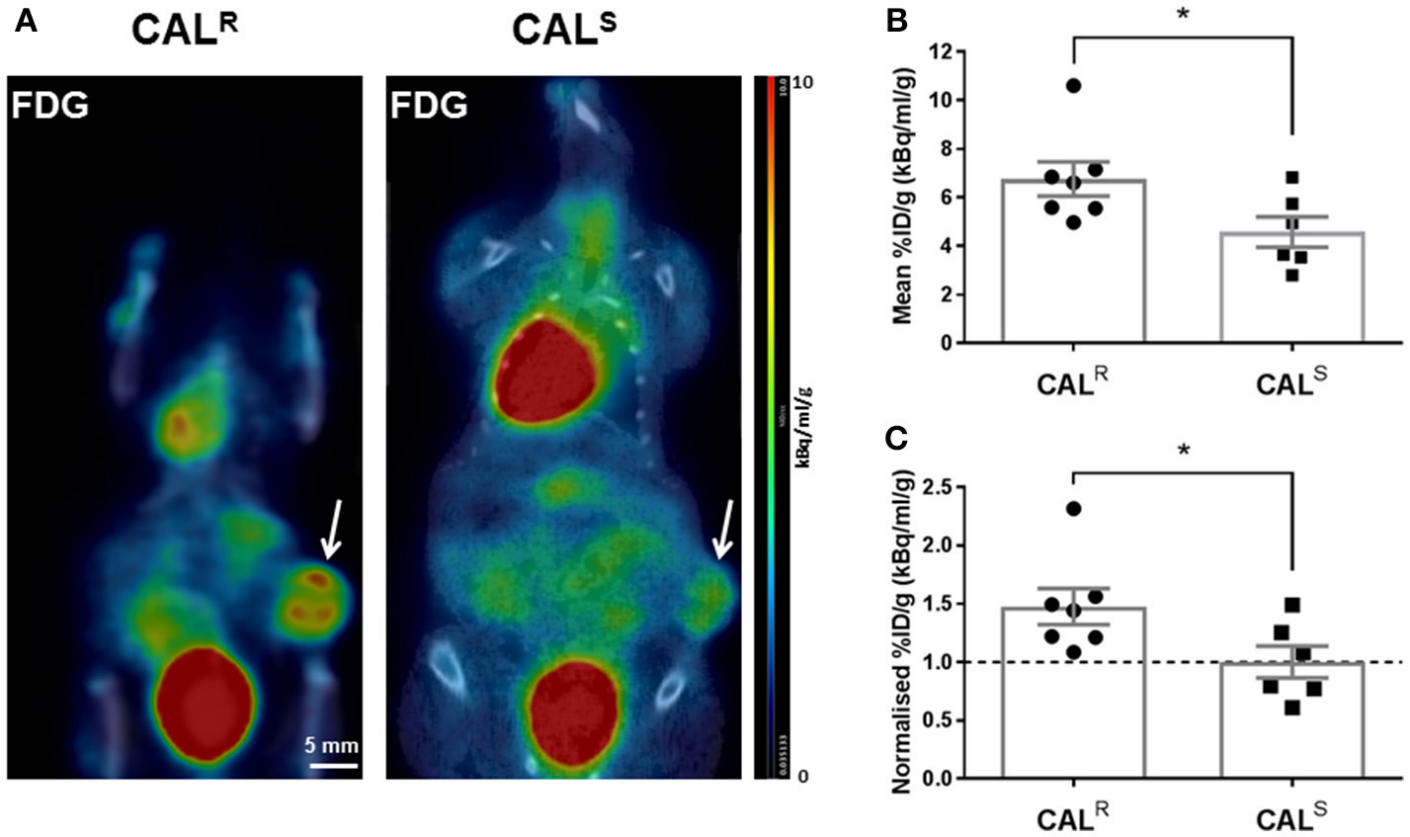
**(A)** Coronal whole body PET-CT images of mice bearing CAL^R^ and CAL^S^ HNSCC xenografts (arrowed) acquired 1 h post injection of ^18^F-FDG. The images are shown on the same PET scale of 0–10% injected dose per gram (%ID/g). Marked uptake of ^18^F-FDG was also observed in the heart and bladder. **(B,C)** A significantly higher relative uptake of ^18^F-FDG was determined in the CAL^R^ cohort compared to the CAL^S^ tumors. Data are the individual **(B)** %ID/g and **(C)** normalized %ID/g for each tumor (relative to the average of the CAL^S^ cohort), and the cohort mean ± 1 s.e.m., *p < 0.05, Student's *t*-test.

Representative whole body PET-CT images acquired from CAL^R^ and CAL^S^ tumor-bearing mice 1 h post injection of ^18^F-FDG are shown in Figure 3A. Greater uptake of ^18^F-FDG was apparent across the CAL^R^ xenografts, resulting in a significantly (*p* < 0.05) higher %ID/g in the CAL^R^ tumors compared to the CAL^S^ (Figure 3B). The relative difference between the CAL^R^ and CAL^S^ xenograft tracer uptake was quantified by normalizing the %ID/g results to the average value found in the CAL^S^ cohort, and also revealed a significant 48% (*p* < 0.05) higher relative uptake of ^18^F-FDG in the CAL^R^ tumors (Figure 3C).

**Figure 4:**
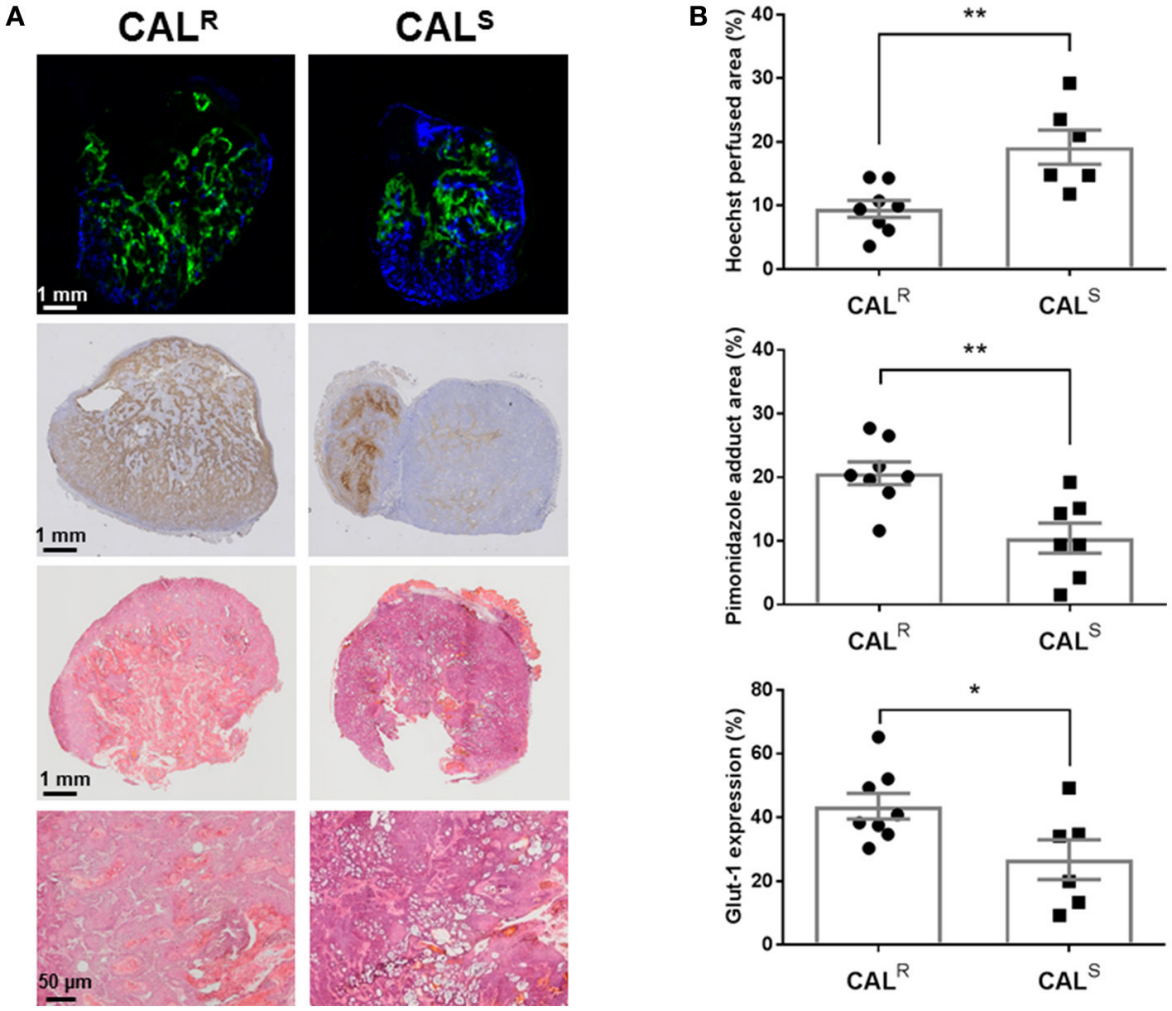
**(A)** Composite fluorescence images of Hoechst 33342 uptake (blue, perfusion) and pimonidazole adduct formation (green, hypoxia), and bright field images of Glut-1 expression (brown) and H&E staining, acquired from whole sections of representative CAL^R^ and CAL^S^ HNSCC xenografts. High magnification (x200) images from H&E stained sections are also shown. **(B)** Summary of quantitative differences in perfused tumor vessels, hypoxia and Glut-1 expression in the CAL^R^ and CAL^S^ tumors. Significantly lower Hoechst 33342 uptake, and significantly greater pimonidazole adduct formation and Glut-1 expression, was found in the CAL^R^ tumors relative to the CAL^S^ cohort. Data are the individual measurements of Hoechst perfused area (%), pimonidazole adduct area (%) and Glut-1 expression (%) for each tumor, and the cohort mean ± 1 s.e.m., **p < 0.01, *p < 0.05, Student's *t*-test.

Composite fluorescence images of Hoechst 33342 and pimonidazole adducts, and bright field images of Glut-1 expression, in whole sections from CAL^R^ and CAL^S^ tumors, are shown in Figure 4A. Spatially, uptake of the perfusion marker Hoechst 33342 was primarily associated with the periphery of both tumor types, with hypoxic regions, as revealed by pimonidazole adduct formation, more heterogeneously distributed and predominantly located in the tumor core. Abundant Glut-1 expression was apparent across the CAL^R^ tumors, but more localized in the CAL^S^ xenografts. Quantitation of the pathology showed significantly (*p* < 0.01) lower perfusion, and significantly greater hypoxia (*p* < 0.01) and Glut-1 expression (*p* < 0.05) in the CAL^R^ tumors relative to the CAL^S^ cohort (Figure 4B). H&E staining revealed abundant vesicle formation in both the CAL^R^ and CAL^S^ tumors (Figure 4A). There was no significant difference in cellular density between the two tumor types [CAL^R^ 45 ± 3 nuclei/0.01 mm^2^ (*n* = 7), CAL^S^ 49 ± 3 nuclei/0.01 mm^2^ (*n* = 5)].

## Discussion

Resistance is one of the major reasons for the failure of targeted cancer drugs. The initial response and subsequent relapse of HNSCC patients treated with EGFR TKIs is well documented (13, 14). Methods that can accurately predict which HNSCC patients will benefit from EGFR-targeted therapies, and inform on response, would positively impact on treatment planning in this patient population. To this end, we evaluated MRI and PET-derived imaging biomarkers in CAL^R^ and CAL^S^ HNSCC xenografts to identify and characterize any phenotypic differences *in vivo* associated with differential sensitivity to EGFR TKIs (33).

Tumor hypoxia is a well-established cause of treatment resistance, adversely affects the prognosis of HNSCC (21, 22), and moderates the expression and activation of EGFR (26). Both intrinsic susceptibility and oxygen-enhanced MRI are being actively exploited for spatially mapping tumor hypoxia *in vivo* (36, 45, 48–50). Oxygen inhalation induced a reduction in ${\text{R}}_{2}{}^{*}$ of both CAL^R^ and CAL^S^ xenografts, a consequence of a reduction in paramagnetic deoxyhaemoglobin in perfused tumor blood vessels. The reduction in ${\text{R}}_{2}{}^{*}$ was significantly smaller in the more aggressive CAL^R^ xenografts, consistent with their having relatively more impaired haemodynamic (functional) vasculature, and was confirmed histologically by their significantly lower uptake of Hoechst 33342. Hyperoxia increased R_1_ in both CAL^R^ and CAL^S^ xenografts to a similar extent reported in other tumor models (36, 48, 49, 51, 52). The low magnitude of this response suggests that these HNSCC xenografts are largely refractory to hyperoxia-induced changes in R_1_ and hence hypoxic (36, 48, 49). There was no relationship between $\Delta {\text{R}}_{2}{}^{*}$ and ΔR_1_ across the CAL^R^ and CAL^S^ tumors. As expected, extensive hypoxia was histologically evident in both CAL^R^ and CAL^S^ HNSCC xenografts, with significantly greater pimonidazole adduct formation determined in the CAL^R^ tumors. These data suggest that diminished hyperoxia-induced $\Delta {\text{R}}_{2}{}^{*}$ is associated with a more drug resistant phenotype, and re-iterates the contribution of hypoxia in exacerbating resistance to anti-EGFR therapies (26, 27).

The vascular patency and cellularity of CAL^R^ and CAL^S^ xenografts was interrogated using DCE- and DW-MRI respectively. The DCE-MRI biomarkers K^trans^, IAUGC_60_ and EF all indicated significantly lower vascular permeability/perfusion in the more hypoxic CAL^R^ xenografts, aligning with the lower hyperoxia-induced $\Delta {\text{R}}_{2}{}^{*}$ response and reduced Hoechst 33342 uptake seen in these tumors. The CAL^R^ phenotype is consistent with clinical data showing that DCE-MRI estimates of perfusion inversely correlate with pimonidazole adduct formation, and that relatively high pre-treatment K^trans^ is associated with good response, in patients with HNSCC (53, 54). Whilst reduced tumor perfusion/permeability measured by DCE-MRI may thus be indicative of resistance to EGFR antagonists, it also reflects diminished drug delivery and hence response. DW-MRI revealed no significant difference in ADC between the CAL^R^ and CAL^S^ xenografts, and H&E staining confirmed no overall difference in cellular density. A recent study in HNSCC patients showed that pre-treatment ADC was unable to distinguish between eventual responders and non-responders to chemotherapy (55). Discrete areas of elevated ADC were detectable in both CAL^R^ and CAL^S^ xenografts, consistent with vesicle formation previously ascribed to the CAL 27 model (56).

As with many aggressive tumors, HNSCCs exhibit high rates of glycolysis to fulfill their energy requirements (57, 58). ^18^F-FDG PET has been widely used to visualize increased glycolysis in HNSCC *in vivo* (59). Accordingly, ^18^F-FDG uptake was clearly detected in both CAL^R^ and CAL^S^ xenografts. Significantly greater uptake of ^18^F-FDG was determined in the CAL^R^ tumors, and associated with greater Glut-1 expression, as determined by IHC. Elevated levels of lactate have also been reported in CAL^R^, relative to CAL^S^, tumors (60). Together these data are consistent with EGFR TKI-resistance being associated with a switch to a more glycolytic metabolism *in vivo*. Tumor adaptation to treatment with EGFR antagonists thus involves metabolic reprogramming in HNSCC cells, enabling them to survive *in vivo* despite an impaired functional vasculature and hypoxic microenvironment.

Collectively, the data herein suggest that a multi-modal, multiparametric MRI/PET imaging strategy, incorporating intrinsic susceptibility and DCE-MRI with ^18^F-FDG PET, may be useful in correctly identifying response/resistance to TKIs in patients with HNSCC. Such integrated approaches are already being used clinically to assess the degree and spatial distribution of perfusion, hypoxia and metabolism in HNSCC, and are being facilitated by new hybrid PET/MR technology and associated data fusion (61–63). Incorporation of quantitative ${\text{R}}_{2}{}^{*}$ measurements using intrinsic susceptibility MRI into clinical scanning protocols is relatively straightforward, and tumor ${\text{R}}_{2}{}^{*}$ is being actively investigated as an exploratory imaging biomarker of hypoxia in patients with HNSCC (64).

Whilst this study has focused on characterizing the radiological phenotype associated with resistance to EGFR TKIs in xenografts, the same array of imaging biomarkers may inform on tumor response in patients with HNSCC. In support of this, several pre-clinical imaging investigations have reported improved tumor oxygenation (65), perfusion/permeability and water diffusivity (66, 67), and reduced ^18^F-FDG uptake (68), following treatment with gefitinib monotherapy or in combination with chemo/radiation (69).

In summary, we have shown that xenografts established from HNSCC cells resistant to EGFR TKIs are more hypoxic, poorly perfused and more glycolytic than those sensitive to EGFR inhibitors, and that these differences can be visualized and quantified non-invasively *in vivo* using functional MRI and PET imaging. These imaging techniques are either routinely being used in the clinic or are under active development, so adoption of a multi-modal, multiparametric imaging strategy to assess HNSCC response and resistance to EGFR inhibition would enhance expedient treatment selection and scheduling for individual patients.

## Author contributions

LB and CB: study concepts. LB, AS, JP, JB, CB, and SR: study design. LB, AS, JP, and GB: data acquisition. LB, JP, JB, EL, YJ, and TS: quality control of data and algorithms. LB, AS, JP, JB, EL, YJ, TS, CB, and SR: data analysis and interpretation. LB, JB, and SR: statistical analysis. LB, JP, JB, CB, and SR: manuscript preparation. LB, AS, JP, JB, EL, GB, YJ, TS, GK-M, ML, and SE: manuscript editing. CB and SR: manuscript review.

### Conflict of interest statement

The authors declare that the research was conducted in the absence of any commercial or financial relationships that could be construed as a potential conflict of interest.
